# Transcriptomic profiling reveals host-specific evolutionary pathways promoting enhanced fitness in the plant pathogen *Ralstonia pseudosolanacearum*

**DOI:** 10.1099/mgen.0.001142

**Published:** 2023-12-08

**Authors:** Rekha Gopalan-Nair, Marie-Françoise Jardinaud, Ludovic Legrand, Céline Lopez-Roques, Olivier Bouchez, Stéphane Genin, Alice Guidot

**Affiliations:** ^1^​ LIPME, Université de Toulouse, INRAE, CNRS, Castanet-Tolosan, France; ^2^​ INRAE GeT-PlaGe, Genotoul, Castanet-Tolosan, France

**Keywords:** experimental evolution, pathogen adaptation, *Ralstonia pseudosolanacearum*, transcriptomic variation

## Abstract

The impact of host diversity on the genotypic and phenotypic evolution of broad-spectrum pathogens is an open issue. Here, we used populations of the plant pathogen *Ralstonia pseudosolanacearum* that were experimentally evolved on five types of host plants, either belonging to different botanical families or differing in their susceptibility or resistance to the pathogen. We investigated whether changes in transcriptomic profiles, associated with or independent of genetic changes, could occur during the process of host adaptation, and whether transcriptomic reprogramming was dependent on host type. Genomic and transcriptomic variations were established for 31 evolved clones that showed better fitness in their experimental host than the ancestral clone. Few genomic polymorphisms were detected in these clones, but significant transcriptomic variations were observed, with a large number of differentially expressed genes (DEGs). In a very clear way, a group of genes belonging to the network of regulation of the bacterial virulence such as *efpR*, *efpH* or *hrpB*, among others, were deregulated in several independent evolutionary lineages and appeared to play a key role in the transcriptomic rewiring observed in evolved clones. A double hierarchical clustering based on the 400 top DEGs for each clone revealed 2 major patterns of gene deregulation that depend on host genotype, but not on host susceptibility or resistance to the pathogen. This work therefore highlights the existence of two major evolutionary paths that result in a significant reorganization of gene expression during adaptive evolution and underscore clusters of co-regulated genes associated with bacterial adaptation on different host lines.

## Data Summary

The authors confirm all supporting data, code and protocols have been provided within the article or through supplementary data files.

**Impact Statement**

Studies on the process of adaptive evolution of broad-spectrum pathogens to their host have so far mainly focused on the role of genetic changes. Here, we studied in more detail the changes in transcriptome profiles that could occur during the host adaptation process, whether associated with or independent of genetic changes. We used populations of the bacterial plant pathogen *Ralstonia pseudosolanacearum* that were experimentally evolved on five plant species and compared the genomic and transcriptomic profiles between the ancestral and evolved clones. Our results revealed very few, or even no, genetic alterations in the evolved clones, but significant transcriptomic variations were observed, with a large number of differentially expressed genes. We identified groups of virulence genes that were deregulated in several independent evolutionary lineages and found two major patterns of gene deregulation that were dependent on the plant species on which the bacteria evolved, but not on the susceptibility or resistance of the host to the pathogen. Overall, our analyses highlight the existence of two major evolutionary paths that resulted in a significant rewiring of gene expression during host adaptation and revealed the existence of clusters of co-regulated genes associated with host-specific adaptation.

## Introduction

Understanding how pathogens adapt to their host or to new environmental conditions is still a major concern. Several studies have demonstrated the undeniable role of adaptive mutation fixation across generations to explain adaptation to changing environments or, in the case of pathogens, to new hosts [1, 2]. Changes in gene expression, whether associated with or independent of genetic changes*,* in response to environmental factors also play a major role in the generation of adapted phenotypes [3, 4]. However, very few studies have focused on transcriptomic variation during the host adaptation process.

In a previous work, we conducted an experimental evolution of the plant pathogen *Ralstonia pseudosolanacearum* strain GMI1000 through a serial passage experiment into the stem of various plants [5]. The vast majority of the clones we obtained had significantly higher fitness gains than the ancestral clone. We then performed transcriptomic analyses of 10 clones evolved in the resistant tomato Hawaii 7996, which revealed significant transcriptomic variations and a convergence towards a global rewiring of the virulence regulatory network [6]. As the evolution experiment was performed on several plant species [5], we sought to determine the extent to which the transcriptional profiles mobilized for adaptation to tomato Hawaii were conserved for adaptation to other plants. The underlying questions were (i) whether the adaptation process results in a convergence of responses at the transcriptional level and (ii) whether the host plant species has an effect on this transcriptional reorganization.

We thus investigated genomic and transcriptomic variations in 21 additional clones obtained by experimental evolution of strain GMI1000 in either susceptible (tomato *var*. Marmande, eggplant *var*. Zebrina) and tolerant (bean *var*. blanc précoce, cabbage *var*. Bartolo) hosts.

## Methods

### Bacterial strains and growth conditions

The GMI1000 strain and a list of 21 evolved clones, derived from GMI1000, were investigated in this study (Table 1). The evolved clones were generated in our previous work by serial passage experiments (SPE) of the GMI1000 strain in 4 different plant species during 300 bacterial generations [5]. Five biological SPE replicates (named A, B, C, D and E) were conducted in parallel for each plant species, generating five lineages of evolved clones per plant species (Table 1). For each evolved clone, the adaptive advantage was compared to the ancestral clone through the measure of their competitive index (CI) during *in planta* competition assays [5, 6].

**Table 1.** List of evolved clones with their genomic polymorphisms and transcriptomic variations of known virulence determinants

Experimental host	Lineage	Evolved clone	Mean CI	Mutations			No. of DEGs (I logFC I > 1; *P*-value FDR <0.05)	Varying expression of known virulence determinants														Reference
								*efpR*	*efpH*	*hrpB*	*prhP*	*solI*	*solR*	*rasI*	*rasR*	*rsl2*	*lecM*	*narJ*	*narK1*	*nasF*	*nosZ*	
Tomato *var*. Marmande (suceptible host)	A	Mar26a1^∗^	5.6	*RSc2508* ^*IS, -120*^	tktA^*R326G*^	RSp0128-0154^*Del 33kb*^	1390		Up (4.43)			Down (−2.75)	Down (−2.92)	Up (2.07)		Down (−8.50)		Up (3.03)	Up (2.99)	Up (3.19)	Up (3.24)	[5], this study
	A	Mar26a2	5.4	*RSc2508* ^*IS, -120*^			1424		Up (4.38)			Down (−3.22)	Down (−3.21)	Up (2.20)		Down (−9.28)		Up (2.98)	Up (2.73)	Up (3.20)	Up (3.08)	[5], this study
	B	Mar26b2^∗^	3.9	*phcS* ^*T26M*^			2368		Up (4.10)			Down (−2.40)		Up (2.05)		Down (−2.79)	Down (−4.45)	Up (1.92)	Up (2.81)	Up (3.18)	Up (4.96)	[5], this study
	D	Mar26d2	5.7	*RSc2508* ^*IS, -120*^			1387	Down (−2.24)	Up (4.00)							Down (−9.57)		Up (2.69)	Up (2.43)	Up (2.87)	Up (2.59)	[5], this study
	E	Mar26e1^∗^	3.4	*RSc2508* ^*IS, -120*^			2174	Down (−2.35)	Up (3.66)		Down (−2.25)	Down (−2.56)	Down (−4.32)		Up (1.97)	Down (−10.6)	Down (−2.13)	Up (2.58)		Up (2.43)	Up (1.87)	[5], this study
	E	Mar26e3	6.3	*RSc2508* ^*IS, -120*^	*RSp1466* ^*In 8 nt,* -*256*^		1371		Up (4.10)			Down (−2.54)	Down (−3.75)	Up (2.20)		Down (−9.16)		Up (2.92)	Up (2.55)	Up (2.98)	Up (2.85)	[5], this study
Eggplant *var*. Zebrina (suceptible host)	B	Zeb26b1	2.7	*RSp0083* ^*IS, 1*^			332							Up (1.88)								[5], this study
	B	Zeb26b5	3.7				239	Down (−1.30)								Down (−1.56)				Up (1.41)		[5], this study
	C	Zeb26c2	2.1	*RSp0127* ^*F91L*^			92															[5], this study
	C	Zeb26c3	1.6	*RSp0127* ^*F91L*^			335			Down (−1.06)						Down (−1.00)				Up (1.27)		[5], this study
	C	Zeb26c4	2.1	*dld* ^*R135S*^			25															[5], this study
	D	Zeb26d1	0.9*ns*				353			Down (−1.00)												[5], this study
	E	Zeb26e1	3.6				515				Down (−1.28)	Down (−2.97)	Down (−3.65)	Up (1.46)		Down (−11.3)						[5], this study
Bean *var*. Blanc Précoce (tolerant host)	A	Bean26a4	6.1	*RSc2508 ^A394^*	*rpo^BD428Y^*		1952					Down (−3.53)	Down (−4.43)		Up (2.38)	Down (−10.5)	Down (−2.59)					[5], this study
	A	Bean26a5	6.5	*RSc2508^A394(-)†^*			1940					Down (−3.53)	Down (−5.06)		Up (2.44)	Down (−10.8)	Down (−2.59)					[5], this study
	C	Bean26c1^∗^	6.6	*efpR^P93Q^*	*purF^G-88A^*		897	Down (−5.95)						Up (2.20)		Down (−3.67)	Down (−3,34)					[5], this study
Cabbage *var*. Bartolo (tolerant host)	B	Cab36b1	4.1	*RSp0955^IS, -1082^*	*flhB^Dup 21 nt, 1129^*		1494	Down (−2.47)	Up (4.44)		Down (−2.36)		Down (−2.06)	Up (2.23)		Down (−6.37)		Up (2.13)	Up (2.24)	Up (3.22)	Up (3.58)	[5], this study
	B	Cab36b2	4.9	*RSc2508^IS, 760^*	*RSp0955^IS, -1082^*	*flhB^Dup 21 nt, 1129^*	2038		Up (4.26)		Down (−2.12)	Down (−2.87)	Down (−3.75)	Up (2.67)		Down (−8.91)		Up (2.79)	Up (2.14)	Up (2.84)	Up (2.45)	[5], this study
	C	Cab36c2	8.8	*spoT^A219P^*	*RSc2428^C-21A^*	*RSc2573-2622^Del 44.4kb^*	1740		Up (4.34)					Up (2.32)				Up (2.84)	Up (2.57)	Up (2.92)	Up (3.30)	[5], this study
	D	Cab36d1	3.5	*phcS^Y106C^*	*flgB^Del 12 nt,483^*	*RSc2573-2622^Del 44.4kb^*	2309		Up (4.94)			Down (−2.38)	Down (−2,64)			Down (−8.35)	Down (−4.43)	Up (2.33)	Up (2.82)	Up (3.64)	Up (4.41)	[5], this study
	E	Cab36e3	9.4	*RSc2573-2622^Del 44.4kb^*			1515		Up (4.31)			Down (−2.34)	Down (−3.59)	Up (2.69)		Down (−9.43)		Up (2.59)	Up (2.73)	Up (2.83)	Up (3.20)	[5], this study
Tomato *var*. Hawaii (resistant host)	A	Haw35a1	8.6	*soxA1^C639R^*			1227	Down (−3,65)			Down (−1.28)	Down (−2.49)		Up (1.87)		Down (−4.46)	Down (−3.42)			Up (1.52)		[6]
	A	Haw35a4	7.2				187							Up (1.38)						Up (1.08)		[6]
	B	Haw35b1	6.5	*RSp1574^V95L^*			478							Up (2.17)		Down (−2.44)	Down (−1.58)		Up (1.56)	Up (1.60)		[6]
	B	Haw35b4	12.9	*RSp1574^V95L^*	*prhP^IS, -6^*		503			Down (−1.38)	Down (−6.36)			Up (2.26)		Down (−1.80)				Up (1.59)		[6]
	C	Haw35c1	4.2				902	Down (−1,91)		Down (−1.68)		Down (−1.62)	Down (−1.12)	Up (2.21)		Down (−4.86)	Down (−1.01)		Up (1.52)	Up (1.16)		[6]
	C	Haw35c2	4.0				272			Down (−1.56)				Up (2.13)						Up (1.41)		[6]
	D	Haw35d3	5.4				125							Up (1.57)						Up (1.30)		[6]
	D	Haw35d5	4.1				269							Up (1.40)		Down (−0.98)				Up (1.38)		[6]
	E	Haw35e1	3.8	*RSp1136^C-218A^*			245							Up (1.28)						Up (1.00)		[6]
	E	Haw35e3	5.4	*RSp1136^C-218A^*	*RSc3094^R162R^*		212							Up (1.53)						Up (1.17)		[6]

The name of the evolved clone indicates the plant species, the number of serial passages, the lineage and the number of the clone. The competitive index (CI) value is indicated for each evolved clone and was measured *in planta* in competition with the ancestral GMI1000 clone in our previous works [5, 6]. In the mutation column, the gene ID or gene name and the modification type are indicated. For SNPs inside the coding sequence, the protein modification is indicated with the original amino acid followed by the position of the SNP and by the new amino acid. For SNPs upstream of the start codon of a gene, the original nucleotide is indicated followed by the position of the SNP from the start codon and by the new nucleotide. For small insertion (In), deletion (Del) and duplication (Dup), the size of the modification is indicated, followed by the position of the modification. For IS insertion (IS), the position of the insertion is indicated upstream of the start codon or in the coding sequence of the gene. For the varying expression of known virulence determinants, the log fold change (logFC) value is given in parentheses. Genes targeted by both mutation and transcriptomic deregulation are highlighted in yellow.

*Genomes of these clones were established with Illumina-seq technology in [5]. DEGs, differentially expressed genes.

†Single-nucleotide deletion. ns, not significantly different from the ancestral clone; nt, nucleotides.

The bacterial strains were revived from −80 °C glycerol collections on agar plates containing BG medium supplemented with d-glucose (5 g l^−1^) and triphenyltetrazolium chloride (0.05 g l^−1^) at 28 °C [7]. For DNA and RNA extractions, bacterial strains were grown in MP synthetic liquid medium supplemented with l-glutamine (10 mM) and oligoelements (1000 mg l^−1^) at 28 °C under agitation at 180 r.p.m. [7]. The pH of the MP medium was adjusted to 6.5 with KOH. The composition of the oligoelements solution is given in the supplementary data file S1, available in the online version of this article.

### Genome sequencing and detection of genomic modifications

DNA were extracted from bacterial cultures in MP medium supplemented with 10 mM glutamine and collected at the beginning of stationary phase (optical density around 1), in similar conditions as for Hawaii clones [6]. Briefly, 20 ml of the bacterial culture was centrifuged at 5000 r.p.m. for 10 min followed by washing the pellets with water and centrifuged again. The pellets were stored at −80 °C until DNA extraction. The DNA were prepared based on the protocol described for high-molecular-weight genomic DNA [8]. DNA concentration and quality were measured by spectrometry using the nanodrop (Thermo Fisher Scientific) and fluorometry using the Qubit dsDNA HS Assay kit (Life Technologies).

DNA sequencing was performed at the GeT-PlaGe core facility, INRAE Toulouse, France, and at Gentyane core facility, INRAE Clermont-Ferrand, France, in similar conditions as for Hawaii clones [6]. Both Illumina and Pacbio sequencing technologies were used for the detection of single-nucleotide polymorphisms (SNPs) or small insertion–deletions (InDels) and for the detection of large genomic rearrangements, respectively. Briefly, for Illumina sequencing, DNAseq libraries were prepared according to Bioo Scientific’s protocol using the Bioo Scientific PCR free Library Prep kit (PerkinElmer) and sequenced on an Illumina Miseq using a paired-end read length of 2×150 bp with the Illumina Miseq Reagents micro V2 kits (Illumina). For PacBio sequencing, SMRTbell libraries of multiplex samples were performed according to the manufacturer’s instructions ‘Procedure-Checklist-Preparing-Multiplexed-Microbial-SMRTbell-Libraries-for-the-PacBio-Sequel-System’ and sequenced on SMRTcells on the Sequel1 instrument at 6 pM with 120 min pre-extension and 10 or 20 h movies using Sequencing Primer V4, polymerase V3, diffusion loading. Detection of genomic modifications was performed as described in our previous work [6]. All detected genomic modifications were checked by PCR amplification and sequencing with the Sanger technology using the primers reported in Table S1.

### RNA extraction and RNA sequencing

RNA were extracted from the same bacterial cultures prepared for DNA extraction in MP medium supplemented with 10 mM glutamine and collected at the beginning of stationary phase (optical density around 1). This condition was preferred to the *in planta* environment to avoid biases associated with bacteria extraction and sequencing. MP medium with glutamine was used to mimic the xylemic environment of the plant, with glutamine being the main compound of xylem sap in most plant species [9]. RNA extraction was performed in similar conditions as for Hawaii clones [6]. Briefly, growth in 20 ml of the bacterial culture was stopped by the addition of 1 ml ethanol/phenol (95 : 5) and mixed well by vortexing for 1 min. The mixture was then centrifuged at 10000 r.p.m. for 20 min at 4 °C and the pellets were stored at −80 °C until RNA extraction. Total RNA was extracted and ribosomal RNAs were depleted as previously described [10]. Three biological replicates were conducted for each of the 21 clones and the GMI1000 ancestor strain.

Oriented paired-end RNA sequencing (RNAseq) was performed at the GeT-PlaGe core facility, INRAE Toulouse, France. The 63 RNAseq libraries were prepared according to Illumina’s protocols using the Illumina TruSeq Stranded mRNA sample prep kit (Illumina) to analyse mRNA. RNA were then fragmented to generate double-stranded cDNA and adaptators were ligated to be sequenced. A total of 11 cycles of PCR were applied to amplify libraries. Library quality was assessed using a fragment analyser (Agilent) and quantification of the libraries was performed by qPCR using the Kapa Library Quantification kit (Kapa). RNAseq experiments were performed on four lanes of an Illumina HiSeq3000 using a paired-end read length of 2×150 bp with the Illumina HiSeq3000 sequencing kits (Illumina).

### Mapping and analysis of RNAseq data

RNAseq read pairs were mapped onto the *R. pseudosolanacearum* GMI1000 genome using Glint v1.0 rc12 software (https://forge-dga.jouy.inra.fr/projects/glint) with parameters set as follows: matches ≥75 nucleotides, ≤4 mismatches, no gap allowed, only best scoring hits considered. Ambiguous matches (same best score) were removed.

Differentially expressed genes (DEGs) were detected with EdgeR Bioconductor package version 3.30.3 [11]. Normalization was performed using the trimmed mean of M-values (TMM) method [12]. Quality control plots of normalized data sets and reproducibility of biological repeats were generated by principal component analysis using the factoMineR version 2.8 package [13]. The correlation between the biological replicates for each clone was estimated by calculating the correlation coefficient of Spearman. Differences and similarities in gene expression between clones were tested by calculating the Euclidean distance and shown on heatmaps (package version 1.0.12). DEGs were then called using the generalized linear model (GLM) likelihood ratio test using a false discovery rate (FDR)-adjusted *P*-value <0.05 [14]. The 400 top DEGs in the 31 evolved clones of *R. pseudosolanacearum* (21 clones from the present work and 10 clones from our previous work [6]) were selected and ternary encoded (−1,0,1) according to their deregulation. Spearman correlation coefficients were calculated using the corr function from the stats R package (version 4.4.2) on all combinations of the 31 subsets of ternary-encoded DEGs and the package corrplot (version 0.92) allowed us to construct the correlation matrix. PERMANOVA was conducted to compare the ternary-encoded 400 top DEGs using permutational multivariate analysis of variance (PERMANOVA – vegan package version 2.6–4) and the package PairwiseAdonis (version 0.4.1) executed multilevel pairwise comparison with Bonferroni-adjusted *P*-values. Selection of DEGs in at least one of the evolved clones from each plant species allowed the construction of five subsets, which were used for Venn diagram representation (package ggVennDiagram version 1.2.3)

Double hierarchical clustering analysis of all the ternary-encoded 400 top DEGs was performed using the hclust function from the stats R package (version 4.3.0), which computed Euclidean distances between clone profiles. Clustering was computed with the Ward’s D2 method and a heat map was generated with the gplots R package (version 3.1.3).

## Results and discussion

We first investigated the genomic variations in the 21 clones compared to their ancestor using whole-genome sequencing (Table 1) . These analyses revealed between zero and three genomic polymorphisms per clone compared to the ancestor. This low number of mutations is in the range of what we found in our previous sequenced evolved clones [5, 6] and included either SNPs, indels, duplication, IS insertion or large deletion (Table 1). All these genomic polymorphisms were confirmed by PCR amplification followed by Sanger sequencing or gel electrophoresis.

We then investigated the transcriptomic variations in the 21 evolved clones compared to their ancestor using the RNA-sequencing approach. Analysis of RNAseq data revealed that all samples rendered between 0.7 and 1.3 million GMI1000-mapped reads. Statistical analyses demonstrated good reproducibility between replicates for each clone and showed that all replicates from all the 21 evolved clones were different from the 3 replicates of the GMI1000 ancestral clone. DEGs between the evolved clones and the ancestral clone were considered as those presenting an FDR-adjusted *P*-value (padj,FDR) <0.05 (Tables 1 and S2). In the 21 newly investigated clones, when considering a log fold change of expression I logFC I>1, the number of DEGs varied between 25 and 2368 and was not correlated to the number of mutations (Table 1). A high number of DEGs was detected in the three clones evolved in eggplant Zebrina despite no detected genetic alteration, confirming a previous observation that some transcriptomic variations may be dependent on epigenetic regulation, a phenomenon reported in prokaryotes although still scarcely investigated [6, 15–17].

By looking more precisely at the DEGs, it appeared that there were groups of genes that were deregulated frequently, even recurrently, in several independent evolutionary lineages and regardless of the host considered (Table 1). In agreement with what was observed for the Hawaii clones, we detected a significant downregulation for the virulence gene regulators *efpR*, *hrpB* and *prhP* [6] in several newly investigated clones. In addition, we observed a significant upregulation for the *efpH* gene, a virulence regulatory gene homologous to *efpR* [18], but exclusively in clones evolved in tomato Marmande and in cabbage. We also observed significant variations in the expression pattern of genes involved in quorum-sensing-dependent virulence signalling pathways such as *solI/solR* [19] in 14 clones; *rasI*/*rasR* [20] in 24 clones; as well as the lectin encoding genes *lecM* and *rsl2* [21] in 21 clones. Genes involved in the denitrification pathway such as *narJ/narK1* and *nosZ* [22] were exclusively upregulated in the tomato Marmande and cabbage clones and *nasF*, a nitrate transporter protein, was found to be upregulated in 23 clones.

**Fig. 1.**
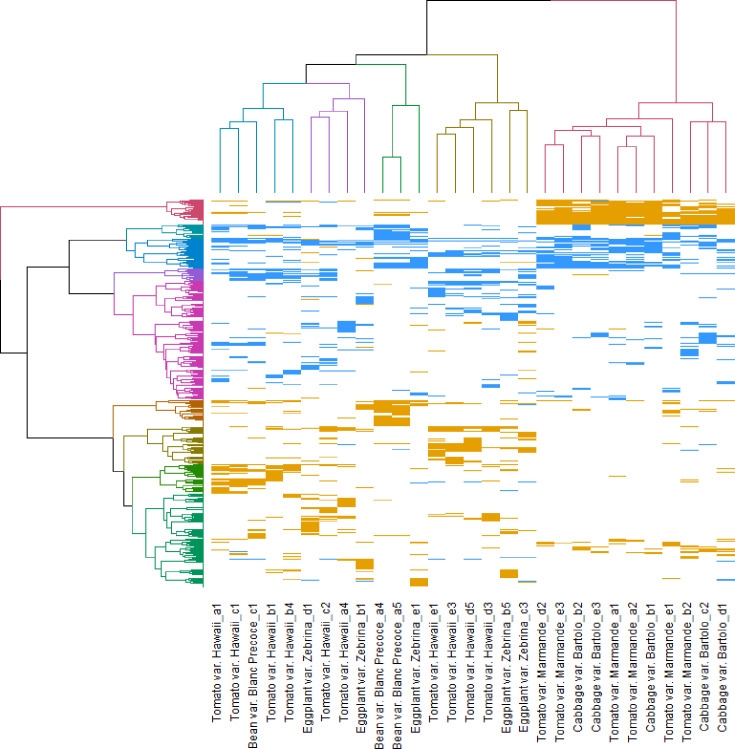
Double hierarchical clustering analysis of the 400 top differentially expressed genes (DEGs) in 31 evolved clones of *R. pseudosolanacearum *. The 400 top DEGs (FDR<0.05) (200 most upregulated and 200 most downregulated) were selected for each clone. Down- and upregulated genes were ternary encoded, respectively −1 (blue), 0 (white) and 1 (orange). The hclust function from the stats R package (version 4.3.0) computed Euclidean distances between clone profiles. Clustering was computed with Ward’s D2 method and a heat map was generated with the gplots R package.

To go further in the analysis and increase its significance, we focused on the 400 top DEGs for each clone (200 most upregulated and 200 most downregulated). No gene in this top list was similarly regulated in all 31 evolved clones. However, when we compared the subsets of DEGs for each plant species (DEG in at least 1 of the evolved clones of each plant species), we found a list of 121 DEGs shared between all plants and from 84 to 506 DEGs that are specific of a plant species (Fig. S1 and Table S3). The comparison of these DEGs using a double hierarchical clustering is presented in Fig. 1. This clustering showed that the evolved clones can be separated into two groups according to the host on which they have evolved, with two main branches clearly distinguishing the clones evolved on tomato Marmande and cabbage on the one hand and the clones evolved on tomato Hawaii, eggplant and bean on the other. These two branches were confirmed by a PERMANOVA test and an analysis of the Spearman correlation coefficient between all 31 evolved clones (Figs S2, S3 and Table S4). This observation suggested that there are at least two major patterns of gene deregulation associated with strain GMI1000 adaptation to its hosts, and that these patterns are associated with the host genotype but not the host susceptibility to bacterial wilt disease. In terms of discriminating genes, it appears that the tomato Marmande/cabbage branch is associated with an upregulated expression of cluster 1 (Fig. 1 and Table S2), which includes 180 genes, such as *efpH*, *narJ* and *nosZ*. The analysis also confirmed downregulation of part of the *hrpB* regulon (cluster 4) in several clones evolved on tomato Hawaii, eggplant and bean, showing significant differences in the expression of several genes encoding type 3 effectors in Hawaii-evolved clones [6].

Altogether, these data highlight a significant reorganization of gene expression associated with adaptation of *R. pseudosolanacearum* to multihost species, which converged toward two major patterns of gene deregulation according to the host genotype. In all cases, a relatively small number of genes (including several transcriptional regulators such as *efpR*, *efpH*, *hrpB*, *rasI*/*R*, *solI*/*R*, *rsl2*) seem to play a key role in these transcriptomic rewirings. The distribution of these regulatory genes in different clusters of co-regulated genes opens avenues for further characterization of these regulons and possible cross-regulations associated with the adaptive process to host plants.

## Supplementary Data

Supplementary material 1

Supplementary material 2
