# Supplementary material for: Transcriptomic profiling reveals host-specific evolutionary pathways promoting enhanced fitness in the plant pathogen Ralstonia pseudosolanacearum
Source: Microb Genom. 2023 Dec 8;9(12):001142. doi: 10.1099/mgen.0.001142 (PMC10763508; doi:10.1099/mgen.0.001142)
Supplement: Supplementary material 1 [file mgen-9-1142-s001.pdf]

Gopalan-Nair et al.

Supplementary material 1

Composition of the oligoelements solution 1000X (250 ml) and protocol

This oligoelement base composition is derived from the Hutner's trace element. The protocol should provide a stable solution, i.e. no salt precipitation, over 4°C storage.

1. Iron solution.

For 250 ml, dissolve the listed salts in 100 ml of distilled water in the order indicated:

$FeSO_4$, 7 H_2O	1.25 g
Na_2EDTA, 2 H_2O	12.50 g

Adjust the pH with KOH 10N until complete dissolution of EDTA and having a golden yellow solution that should be around pH 8.

2. Trace solution.

Dissolve the listed salts in 100 ml of distilled water in the order indicated:

$ZnSO_4$, 7 H_2O	5.50 g
H_3BO_3	2.85 g
$MnCl_2$, 4 H_2O	1.26 g

$CoCl_2$, 6 H_2O	0.40 g
$CuSO_4$, 5 H_2O	0.39 g
$(NH_4)_6Mo_7O_{24}$, 4 H_2O	0.28 g

3. Combining 1 + 2 solutions.

Combine the solution 1 and the solution 2, and readjust the pH to 6.5 using 10N KOH.

Bring the final volume to 250 ml with distilled water.

Sterilize the solution by filtering using 0.22 µm filter.

Store at 4°C.

The Oligo solution is initially bright green, turning purple upon storage. Precipitates should never be formed.

Gopalan-Nair et al.

Supplementary Figure S1

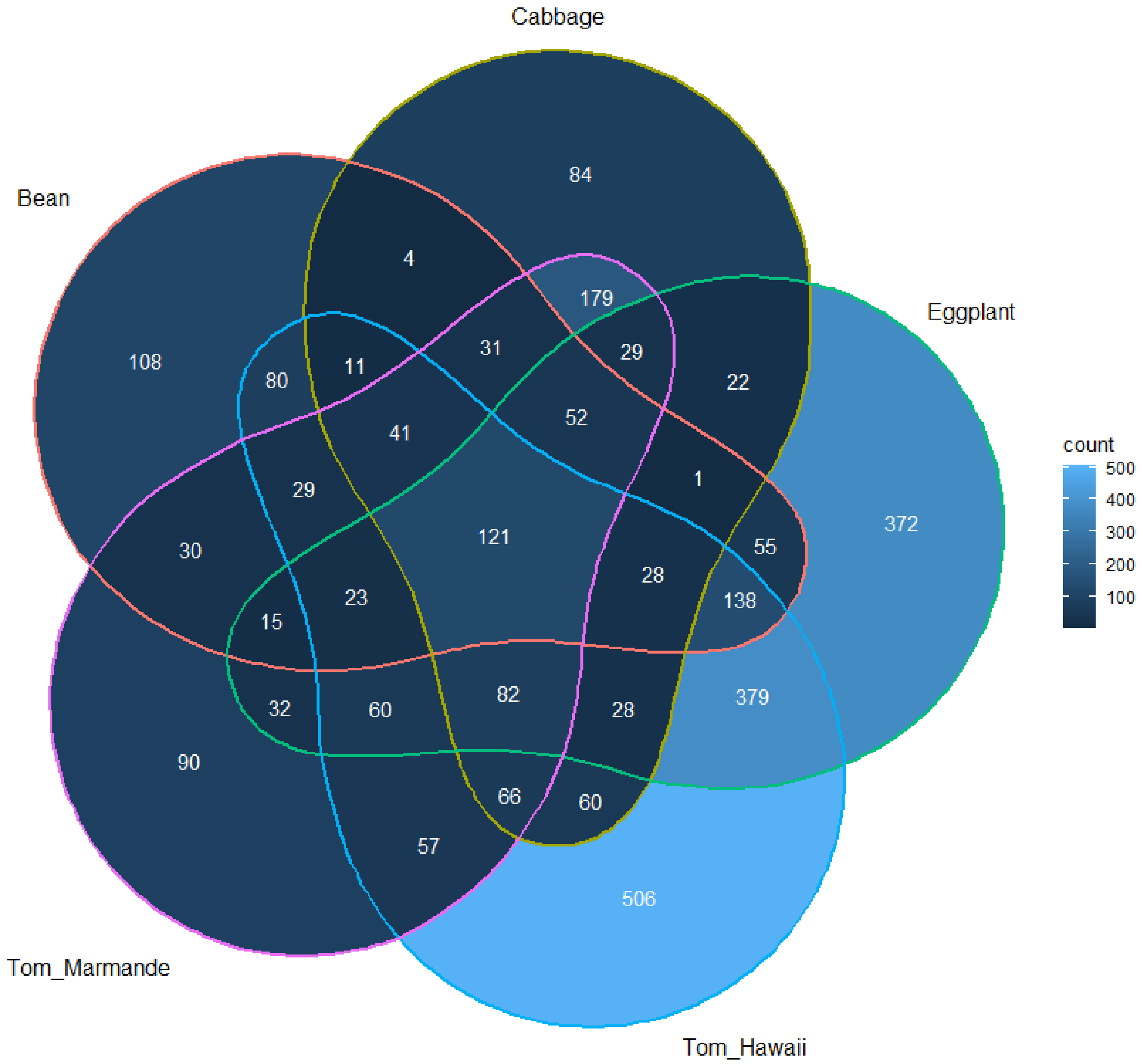

Supplementary Figure S1 Venn diagram illustrating the intersections between the 400 top Differentially Expressed Genes (DEGs) in the 5 plant species.
Using the list of the 400 top DEGs (FDR<0.05) (200 most up-regulated and 200 most down-regulated), subsets were created for each plant species containing DEGs in at least one of the evolved clones. They were used for Venn diagram representation (package ggVennDiagram version 1.2.3). The list of genes specific of a plant species and the list of genes in each intersection are given in Supplementary Table S3.

Gopalan-Nair et al.

Supplementary Figure S2

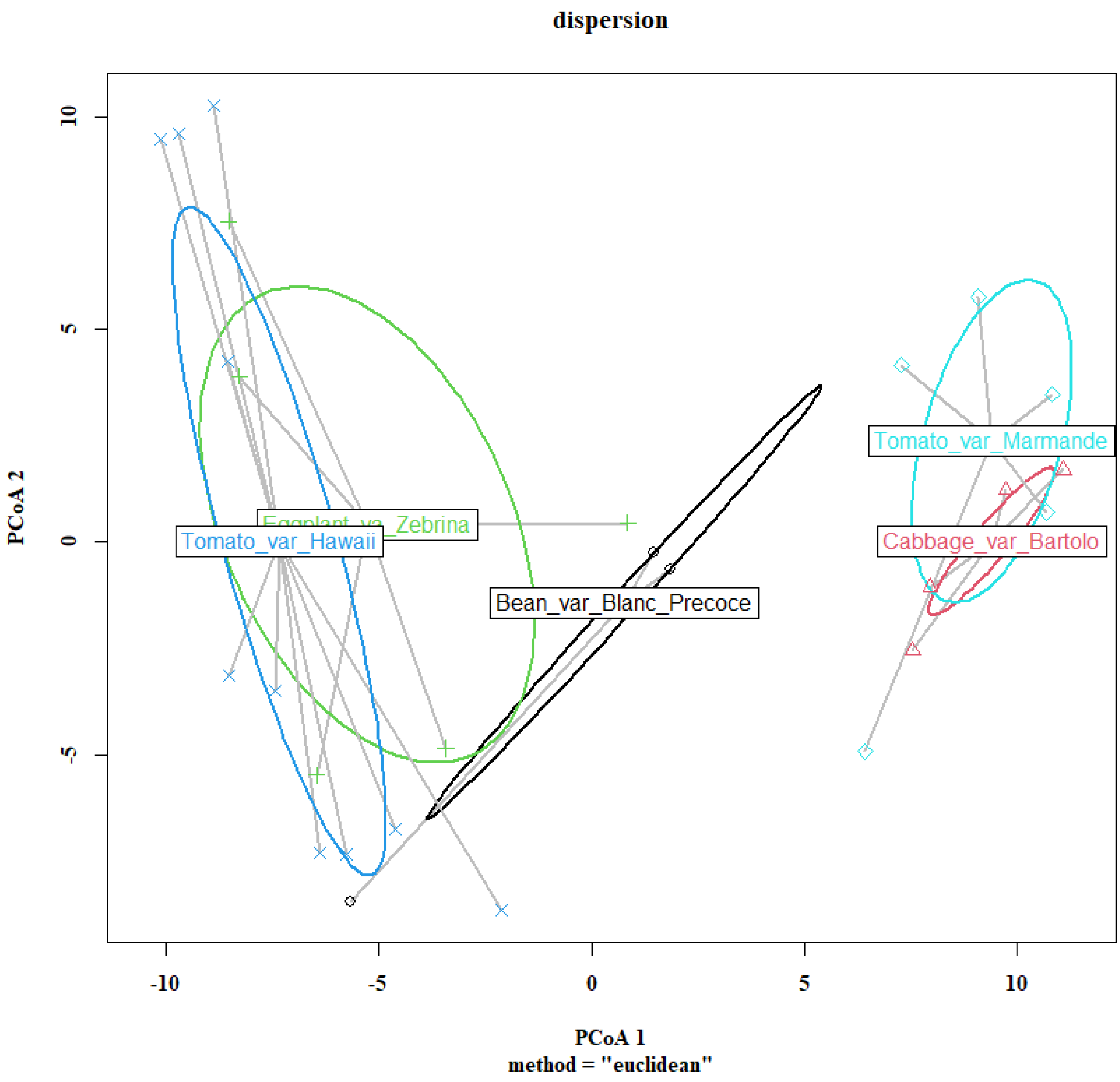

Supplementary Figure S2 Permanova test for similarity based on an Euclidean distance measure between the 31 evolved clones of R. pseudosolanacearum.
The ternary encoded 400 top DEGs (FDR<0.05) (200 most up-regulated and 200 most down-regulated) were compared using Permutational multivariate analysis of variance (PERMANOVA – vegan package version 2.6-4). PairwiseAdonis package (version 0.4.1) allowed multilevel pairwise comparison with Bonferroni adjusted p.values (see Supplementary Table S4 for p.values).

Gopalan-Nair et al.

Supplementary Figure S3

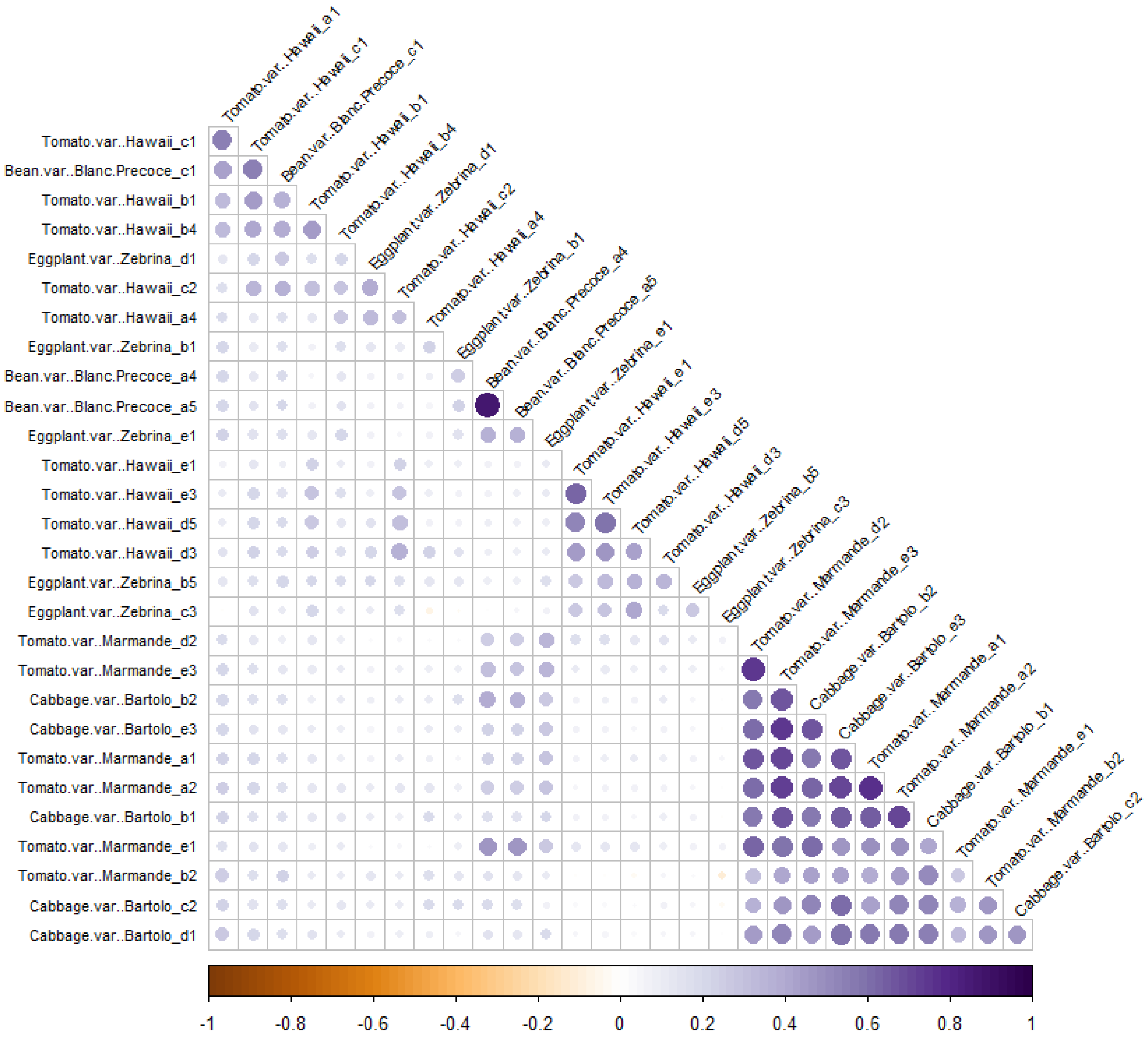

Supplementary Figure S3 Correlation matrix between the 31 evolved clones of R. pseudosolanacearum based on the 400 top Differentially Expressed Genes (DEGs). The 400 top DEGs (FDR<0.05) (200 most up-regulated and 200 most down-regulated) were selected for each clone. Down- and up-regulated genes were ternary encoded, respectively -1, 0 and 1. The corr function from the stats R package (version 4.4.2) computed Spearman correlation coefficient between all 31 clones and the correlation matrix was drawn with the corrplot R package (version 0.92).
